# Archiving and disseminating integrative structure models

**DOI:** 10.1007/s10858-019-00264-2

**Published:** 2019-07-05

**Authors:** Brinda Vallat, Benjamin Webb, John Westbrook, Andrej Sali, Helen M. Berman

**Affiliations:** 1 Institute for Quantitative Biomedicine, Piscataway, USA; 2 RCSB Protein Data Bank, Piscataway, USA; 3 Department of Chemistry and Chemical Biology, Rutgers, The State University of New Jersey, Piscataway, NJ 08854 USA; 4 Department of Bioengineering and Therapeutic Sciences, University of California at San Francisco, San Francisco, CA 94143 USA; 5 Department of Pharmaceutical Chemistry and California Institute for Quantitative Biosciences, University of California at San Francisco, San Francisco, CA 94143 USA; 6 Lead Contacts, San Francisco, USA; 7 Lead Contacts, Piscataway, USA

**Keywords:** Integrative modeling, Hybrid modeling, PDB, mmCIF dictionary, Deposition, Model validation

## Abstract

Limitations in the applicability, accuracy, and precision of individual structure characterization methods can sometimes be overcome via an integrative modeling approach that relies on information from all available sources, including all available experimental data and prior models. The open-source Integrative Modeling Platform (IMP) is one piece of software that implements all computational aspects of integrative modeling. To maximize the impact of integrative structures, the coordinates should be made publicly available, as is already the case for structures based on X-ray crystallography, NMR spectroscopy, and electron microscopy. Moreover, the associated experimental data and modeling protocols should also be archived, such that the original results can easily be reproduced. Finally, it is essential that the integrative structures are validated as part of their publication and deposition. A number of research groups have already developed software to implement integrative modeling and have generated a number of structures, prompting the formation of an Integrative/Hybrid Methods Task Force. Following the recommendations of this task force, the existing PDBx/mmCIF data representation used for atomic PDB structures has been extended to address the requirements for archiving integrative structural models. This IHM-dictionary adds a flexible model representation, including coarse graining, models in multiple states and/or related by time or other order, and multiple input experimental information sources. A prototype archiving system called PDB-Dev (https://pdb-dev.wwpdb.org) has also been created to archive integrative structural models, together with a Python library to facilitate handling of integrative models in PDBx/mmCIF format.

## Overview of integrative structure modeling

Interactions among molecules lead to the emergence of biological phenomena—most evidently in the forms of macromolecular machines and dynamic liaisons that transmit information and control behaviors. Thus, the structures of proteins and their complexes are generally helpful in understanding their function, modulating their activities, and mapping their evolution. Experimental determination of the structures of biomolecular systems is often rather difficult, as no single experimental method is universally applicable. For example, crystals suitable for X-ray crystallography cannot always be produced, especially for large assemblies of multiple components (Blundell and Johnson 1976; Holcomb et al. 2017). Although cryo-electron microscopy (cryo-EM) can be used to study large assemblies, the resolution can be limited (Chiu et al. 2005; Lucic et al. 2008; Stahlberg and Walz 2008). Finally, molecular biology, biochemistry, and proteomics techniques, such as yeast two-hybrid (Parrish et al. 2006), affinity purification (Fernandez-Martinez et al. 2012), and mass spectrometry (Gingras et al. 2007), can yield information about the interactions between proteins, but not the positions of these proteins within the assembly or the structures of the proteins themselves.

Limitations in the applicability, accuracy, and precision of individual structure characterization methods can sometimes be overcome via an integrative modeling approach that relies on information from all available sources, including all available experimental data and prior models (Sali et al. 2003; Ward et al. 2013; Joseph et al. 2017; Kim et al. 2018; Rout and Sali 2019) Integrative modeling is cast as a computational optimization problem in which information can be used in the following five ways, guided by maximizing the accuracy and precision of the model while remaining computationally feasible: (i) representing components of a model with some variables (*e.g.*, atomic coordinates, coarse-grained representations), (ii) scoring alternative models for their consistency with input information, (iii) searching for good-scoring models, (iv) filtering models based on input information, and (v) validation of models. Much of the input information about the modeled system is encoded into data-based restraints comprising a scoring function ((ii) above) used to evaluate candidate models produced by structural sampling ((iii) above). In this regard, integrative modeling is similar to protein structure determination by nuclear magnetic resonance (NMR) spectroscopic methods in which spatial restraints implied by the NMR data, such as nuclear overhauser effects (NOE) and J-coupling constants, must be satisfied. By simultaneously considering all available information, the integrative approach maximizes the accuracy, precision, completeness, and efficiency of structure determination.

Numerous static structures of large complexes have already been solved using integrative methods; for example, the 26S proteasome (Lasker et al. 2012), the type III secretion system needle (Loquet et al. 2012), chromatin comprising the alpha-globin gene neighborhood (Bau et al. 2011), the yeast core spindle pole body (Viswanath et al. 2017a), and the yeast nuclear pore complex (NPC) (Kim et al. 2018). Moreover, the integrative approach can be extended from modeling a single static structure to computing models of multiple structural states in a heterogeneous sample (e.g., the two states in the functional cycle of PhoQ kinase (Molnar et al. 2014)), spatiotemporal models of dynamic processes (e.g., macromolecular transport through the NPC (Raveh et al. 2016; Timney et al. 2016)), and models of molecular networks (e.g., metabolic pathway for gulonate synthesis (Calhoun et al. 2018)).

### Modeling with IMP

**Fig. 1:**
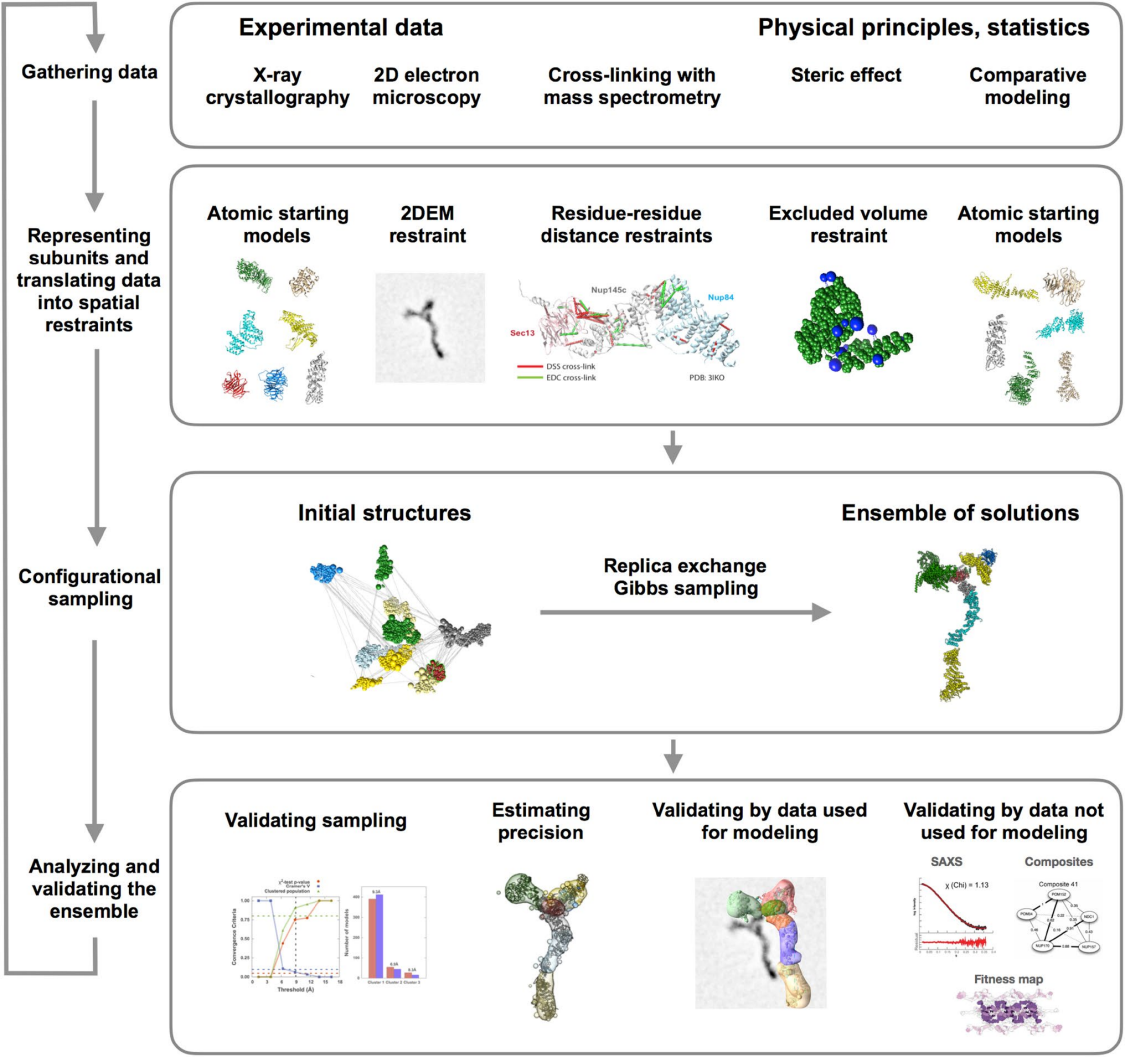
The four-step modeling workflow as implemented in the Integrative Modeling Platform. The workflow is illustrated by its application to structure determination of the Nup84 heptamer (Shi et al. 2014). In this application, crystallographic structures and comparative models are used to represent the seven components of the Nup84 complex. The scoring function incorporates data extracted from CX-MS experiments and 2DEM class average images. The sampling explores both the conformations of the components and their configuration, searching for those assembly structures that satisfy the spatial restraints as accurately as possible. In this case, the result is an ensemble of many good-scoring models that satisfy the input data within acceptable thresholds. The sampling is then assessed for convergence, models are clustered, and evaluated by the degree to which they satisfy the data used to construct them as well as omitted data. The protocol can iterate through the four stages, until the models are judged to be satisfactory, most often based on their precision and the degree to which they satisfy the data. The resulting models are deposited in PDB-Dev (Burley et al. 2017; Vallat et al. 2018) with accession number PDBDEV_ 00000001

There are multiple software packages that can be useful for integrative modeling. The open-source *Integrative Modeling Platform* (IMP) software (https://integrativemodeling.org) (Alber et al. 2007a, b; Russel et al. 2009, 2012; Lasker et al. 2010a; Webb et al. 2018) is our attempt to implement all computational aspects of integrative modeling. The modeling process proceeds through four stages (Fig. 1) (Alber et al. 2007a, 2008a; Russel et al. 2012).

In the first stage, all information that describes the system of interest is collected. This information can include data from various experiments, structural propensities such as atomic statistical potentials (Sippl 1990; Shen and Sali 2006), physical principles such as those encoded in molecular mechanics force fields (Brooks et al. 2009), and other models, such as atomic structures of the subunits in a modeled complex.

In the second stage, a suitable representation of the system is chosen depending on the quantity and resolution of the available information. Different parts of a model may be represented at different resolutions, and a given part of the model may be represented in several different ways simultaneously. Next, information is translated into a set of spatial restraints on the components of the system. For example, in early characterizations of the molecular architecture of the NPC (Alber et al. 2007a, b), atomic structures of the protein subunits were not available, but the approximate size and shape of each protein was known, so each protein was represented as a ‘string’ of connected spheres whose volumes were consistent with the protein size and shape. A simple distance between two proteins can be restrained by a harmonic function of the distance, while the fit of a model into a three-dimensional Electron Microscopy (3DEM) density map can be scored by means of the cross-correlation between the model and experimental densities. Next, the spatial restraints are combined into a single scoring function that ranks alternative models based on their agreement with input information.

In the third stage, alternative models are sampled, using a method such as conjugate gradients, molecular dynamics, Brownian dynamics (Chen and Kim 2004), Monte Carlo (Metropolis and Ulam 1949), and divide-and-conquer message passing (Lasker et al. 2009). This sampling generally generates not a single structure, but an ensemble of models that are as consistent with the input information as possible. There may be many different models that score well if the data are incomplete, or none if the uncertainty of the data is underestimated or the representation does not include appropriate degrees of freedom (e.g., too coarse a representation is used, a flexible subunit is modeled as rigid, or a single-state model is used instead of a multiple-state model). Models produced by sampling can be optionally filtered by some information that cannot be feasibly evaluated many times during sampling (e.g., a match between a model and a two-dimensional Electron Microscopy (2DEM) class average (Velazquez-Muriel et al. 2012)).

In the fourth stage, input information and output structures need to be analyzed to estimate structure precision and accuracy, detect inconsistent and missing information, and to suggest most informative future experiments. Assessment begins with structural clustering of the modeled structures produced by sampling, followed by assessment of the thoroughness of structural sampling, estimating structure precision based on variability in the ensemble of good-scoring structures, quantification of the structure fit to the input information, structure assessment by cross-validation, and structure assessment by data not used to compute it (Viswanath et al. 2017b).

Integrative modeling can iterate through these four stages until a satisfactory model is built. Many iterations of the cycle may be required, given the need to gather more data as well as to resolve errors and inconsistent data.

Integrative modeling problems vary in size and scope. Thus, IMP offers a great deal of flexibility and several abstraction levels as part of a multi-tiered platform. At the lowest level, IMP is designed as a toolkit or set of “building blocks,” providing components and tools to allow method developers to convert data from new experimental methods into spatial restraints, to implement sampling and analysis techniques, and to implement an integrative modeling procedure from scratch, using the C ++ and Python programming languages. IMP is freely available as open source software under the terms of the GNU Lesser General Public License. To allow a community of developers to easily add sources of information, sampling schemes and analysis methods, IMP is structured as a collection of self-contained modules that can be developed and distributed independently.

In IMP, models are encoded as collections of particles, each representing a piece of the system. Depending on the data available, particles can be used to create atomic, coarse-grained, and/or hierarchical representations. It is straightforward to represent a protein at any resolution, from fully flexible atomic models (one particle per atom), to rigid bodies, to coarse-grained models consisting of only one or a few particles for the whole protein. Different parts of the model can be represented differently, as dictated by the available information. Each particle has associated attributes, such as coordinates, radius, residue information, and mass. Likewise, an IMP model can consist of one or more states of the same system (e.g., PhoQ kinase in two functional states (Molnar et al. 2014)) and/or multiple similar systems related via an alignment (Echeverria and Sali 2018).

Candidate IMP models are evaluated by a scoring function composed of terms called spatial restraints, each of which measures how well a model agrees with the information from which the restraint was derived. A restraint encodes what is known about structures in general (*e.g.*, a molecular mechanics force field) or what is known about this particular structure (*e.g.*, a distance restraint from NMR measurement). Thus, a candidate model that scores well is generally consistent with all used information. The precision and accuracy of the resulting model ensemble increases with the amount and quality of information that is encoded in the representation, restraints, sampling, and filtering after sampling. IMP’s growing set of restraints supports small angle X-ray (SAXS) profiles (Schneidman-Duhovny et al. 2011), various proteomics data such as data from affinity co-purifications and yeast two-hybrid experiments (Alber et al. 2008b), EM single particle images, 2DEM class averages (Schneidman-Duhovny et al. 2012; Velazquez-Muriel et al. 2012), and 3DEM density maps (Lasker et al. 2010a, b), most of the NMR spectroscopy-derived restraints (Simon et al. 2010), the CHARMM force-field (Brooks et al. 2009), restraints implied by an alignment with related structures (Sali and Blundell 1993), chemical crosslinking (Erzberger et al. 2014), hydrogen–deuterium exchange (Saltzberg et al. 2017), chromosome conformation capture (Bau et al. 2011), Förster resonance energy transfer (FRET) (Bonomi et al. 2014), a variety of statistical potentials (Shen and Sali 2006), and others. A common and powerful application of IMP involves the combination of information on local inter-particle distances and angles, such as that derived from NMR or crosslinking experiments, with overall shape information, such as that provided from 3DEM density maps (Zeng-Elmore et al. 2014; Luo et al. 2015; Robinson et al. 2015; Kim et al. 2018).

For most applications, the full flexibility of defining a system from the bottom up as sets of particles is unnecessary. IMP provides a higher-level interface called *Python Modeling Interface* (PMI) that allows for a top-down representation of the system, using biological names for protein subunits (Saltzberg et al. 2019). It provides simple mechanisms to set up higher order structure, such as multiple copies of subunits or symmetry-related subsets of the system, at multiple resolutions. It also allows easy setup of the myriad advanced restraints available in IMP. Finally, it provides ready-built protocols and other utilities, for example to generate publication-ready plots. Using PMI, the entire modeling protocol can be described with a set of Python scripts, which are typically deposited, together with the input data and output models, in a publicly available repository, such as GitHub and the Worldwide Protein Data Bank (wwPDB) prototype archive for integrative structures called PDB-Dev (Burley et al. 2017; Vallat et al. 2018); for examples, see references (Algret et al. 2014; Erzberger et al. 2014; Shi et al. 2014; Luo et al. 2015; Robinson et al. 2015; Shi et al. 2015; Chen et al. 2016; Fernandez-Martinez et al. 2016; Wang et al. 2017b). Finally, at the highest abstraction levels, for users with limited programming experience, IMP provides less flexible but more user-friendly applications to handle specific tasks, such as fitting of proteins into a density map of their assembly (Lasker et al. 2009), scoring protein–ligand interactions (Fan et al. 2011), combining multiple SAXS profiles (Spill et al. 2014), comparing a structure with the corresponding SAXS profile (Schneidman-Duhovny et al. 2010, 2013, 2016), or enriching pairwise docking using SAXS data (Schneidman-Duhovny et al. 2016); these functionalities can be accessed through web interfaces, from Chimera (Pettersen et al. 2004), or from the command line.

IMP has been used to produce structural models of more than 30 varied biomolecular systems; for example, a eukaryotic ribosome (Taylor et al. 2009), aryanodine receptor channel (Serysheva et al. 2008), the yeast Mediator complex (Robinson et al. 2015), the Hsp90 chaperonin (Krukenberg et al. 2008), a yeast exosome in multiple states (Shi et al. 2015), the actin-scruin complex (Cong et al. 2008), deoxyribose nucleic acid (DNA) transcription factor II H (TFIIH) (Luo et al. 2015), chromatin (Bau et al. 2011; Tjong et al. 2016), and the NPC and its subcomplexes (Alber et al. 2007b; Fernandez-Martinez et al. 2012, 2016; Kim et al. 2014, 2018; Shi et al. 2014; Upla et al. 2017).

### Requirements for archiving integrative models

To maximize the impact of integrative structures, the coordinates should be made publicly available, at least upon publication, as is already the case for structures based on X-ray crystallography, NMR spectroscopy, and 3DEM maps. Moreover, the associated experimental data and modeling protocols should also be archived, such that both the authors and others can easily reproduce the original results. Finally, it is essential that the integrative structures are validated as part of their publication and deposition, as is already the case for other structures currently archived in the Protein Data Bank (PDB) (Gore et al. 2017; Young et al. 2017).

In recognition of the challenges involved in archiving integrative models, the wwPDB convened an Integrative/Hybrid Methods Task Force workshop in 2014. The IHM Task Force made several recommendations to facilitate the archiving of integrative structural models (Sali et al. 2015). A fundamental requirement is the development of a flexible model representation that allows us to represent ensembles of multi-scale, multi-state, and ordered collections of structural models. The representation should also provide support for spatial restraints derived from diverse types of experimental data obtained from different samples, used as input in the modeling. Another requirement is creating the software infrastructure required for deposition, curation, validation, archiving, and dissemination of integrative structures. The development of a flexible data representation and a prototype system for archiving integrative structural models are discussed in sects. “Standards for archiving integrative models” and “The IHM-dictionary”.

Another recommendation from the Task Force was to build a Federation of structural model and experimental data repositories that interoperate with one another. This requires development of well-aligned data standards and data exchange protocols that enable efficient and automated interoperation. Lastly, the Task Force recommended the creation of methods for evaluating and validating integrative structures so that they can be appropriately used for downstream applications. A reasonable starting point for structure validation is the model assessment process outlined in sect. “Modeling with IMP”. However, much more research effort on the part of the entire community is needed to define the necessary validation criteria and implement them in robust software, eventually leading to a validation pipeline that can be part of the archiving process. Work is currently in progress to build an interoperating network of repositories as well as to develop the validation pipeline for integrative models.

### Multi-method structures in the Protein Data Bank

The PDB is the sole international repository for experimentally-determined 3D atomic structures of biological macromolecules (Berman et al. 2000, 2003). When the resource was first established in 1971, X-ray crystallography was the principal method for determining the structures of these molecules and therefore the PDB archived structures determined from diffraction experiments, initially using X-ray and later from neutron radiation. Over time, the structural biology field grew and newer methods of structure determination using NMR spectroscopy and 3DEM were developed. Simultaneously, the PDB expanded itself to serve the needs of the structural biology community and started archiving structures determined using NMR spectroscopy (Borah et al. 1985) and 3DEM (Henderson et al. 1990). In 2008, the PDB began to require the deposition of structure factors for X-ray structures and the deposition of NMR chemical shifts for NMR structures (wwPDB consortium 2007). BioMagResBank (BMRB (Ulrich et al. 2008)) and Electron Microscopy Data Bank (EMDB (Tagari et al. 2002; Lawson et al. 2016; Patwardhan and Lawson 2016)) have been created independently to archive NMR data and 3DEM maps. The availability of the underlying experimental data made it possible to create better validation standards for the structural models archived in the PDB. The wwPDB consortium (Berman et al. 2007) that manages the PDB archive has recently developed the OneDep system (Young et al. 2017) to provide a unified portal for the deposition of structural models determined using X-ray crystallography, NMR spectroscopy, and 3DEM along with associated experimental data that aids structure validation.

**Fig. 2:**
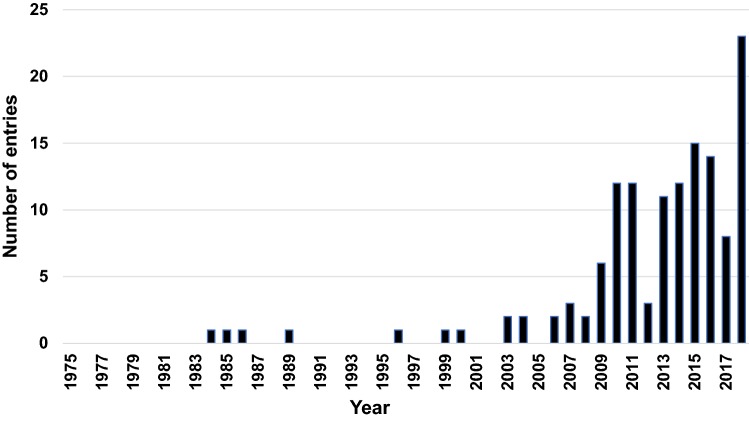
Number of multi-method structures archived in the PDB over the years (data as of December 6, 2018)

In recent times, structural biologists have started to combine data from two or more experimental methods to build structural models of macromolecules. The PDB archives structures determined using multiple methods, where the experiments are carried out on samples of similar composition. Usually, methods capable of resolving atomistic features, such as X-ray crystallography, neutron crystallography, NMR spectroscopy, and 3DEM, can be combined with each other or used in combination with methods that provide coarse-grained information, such as small angle solution scattering (SAS) methods, solid-state NMR spectroscopy, and electron paramagnetic resonance (EPR) spectroscopy. The multi-method experimental structures are distinct from the integrative models where complex computational algorithms combine data obtained from an unrestricted set of experimental observations on a potentially diverse set of experimental samples, although the distinction is more of a degree than kind. Figure 2 shows the historical growth of multi-methods structures in the PDB, which highlights the increase in the deposition of multi-method structures over the last 10 years. Table 1 shows the breakdown of method combinations in multi-methods structures currently released by the PDB. Not surprisingly, multi-method structures in the PDB frequently use X-ray crystallography in combination with neutron diffraction and solution NMR in combination with SAS. To support the facile deposition of structures that use solution NMR in combination with SAS, the wwPDB OneDep team recently extended the deposition infrastructure to handle SAS data. This work has been carried out in collaboration with the SASBDB repository, which archives SAS data (Valentini et al. 2015).

**Table 1:** Combination of methods used to determine multi-method structures currently archived in the PDB and the number of PDB entries with these method combinations (data as of December 6, 2018)

Existing experimental method combinations	Entries released in PDB
X-ray crystallography + solution NMR	1
X-ray crystallography + neutron diffraction	81
X-ray crystallography + solution scattering	2
X-ray crystallography + EPR	7
Solution NMR + solid-state NMR	4
Solution NMR + EM	1
Solution NMR + solid-state NMR + EM	1
Solution NMR + neutron diffraction	1
Solution NMR + solution scattering	17
Solution NMR + EPR	1
Solution NMR + theoretical model	7
EM + solid-state NMR	6
EM + solution scattering	2
EM + solution scattering + solid-state NMR	1
Fiber diffraction + solid-state NMR	1

As more structures were determined by combining data from multiple methods, integrative modelers began exploring the application of additional biophysical techniques such as chemical crosslinking mass spectrometry (CX-MS), SAS, EPR spectroscopy, FRET, hydrogen/deuterium exchange mass spectrometry (HDX-MS), and others, to derive spatial restraints that can be combined to determine structures of complex macromolecular assemblies (Sali et al. 2003; Alber et al. 2007a, b) (Ward et al. 2013; Sali et al. 2015; Rout and Sali 2019). These integrative modeling methods became especially useful to model structures of macromolecular assemblies that are elusive to the traditional methods of structure determination. To adapt to the growing needs of the structural biology community, the PDB, in collaboration with the integrative modeling community, began developing the infrastructure required to archive, validate, visualize, and disseminate integrative structural models.

### Standards for archiving integrative models

A primary requirement for archiving data is the creation of a standard representation of the data to be archived. These data standards provide the foundation for building an archive. Under the auspices of the IUCr, the mmCIF data representation (Fitzgerald et al. 2005) was developed for structures of macromolecules determined using X-ray crystallography. That data dictionary is based on a robust framework that supports the representation of macromolecular structure data and associated metadata. The framework also provides mechanisms to include metadata used for assessing and maintaining data consistency, such as definitions of data types, boundary conditions, controlled vocabularies, and parent–child relationships with other data items.

Extensions of the mmCIF data representation have been created to represent different kinds of experimental data and structural restraints derived from them. These extensions are now embodied in the PDBx/mmCIF dictionary that is the standard for data archiving by the PDB (Westbrook 2013). For example, the NMR community has extended the PDBx/mmCIF dictionary to represent NMR restraints in the NMR Exchange Format (NEF) dictionary (Gutmanas et al. 2015) and the SAS community has created the sasCIF extension dictionary (Malfois and Svergun 2000; Kachala et al. 2016), which is used by the SASBDB repository (Valentini et al. 2015) to archive SAS data. We have extended the existing PDBx/mmCIF data representation to address the requirements for archiving integrative structural models. This extended data representation, called the IHM-dictionary (Vallat et al. 2018), is developed as a collaborative project that is distributed freely through a public GitHub repository (https://github.com/ihmwg/IHM-dictionary). Building an extension dictionary based on the PDBx/mmCIF representation allows us to use a single robust framework to create new definitions while retaining the existing definitions in the main dictionary where applicable. This design helps avoiding duplication while allowing us to focus on the new definitions that address the current requirements.

#### The IHM-dictionary

The IHM-dictionary is an extension of the PDBx/mmCIF dictionary and therefore only defines those terms required for representing integrative structural models that are not already included in the PDBx/mmCIF dictionary. For instance, the nomenclature and chemistry of small molecules, polymeric macromolecules, and molecular complexes consisting of small molecules and macromolecules are already defined in the PDBx/mmCIF dictionary. Similarly, the definitions of the molecular structure in terms of atomic coordinates are also clearly represented in the PDBx/mmCIF dictionary. Although these definitions provide the foundation for representing the chemistry and structure of a molecular system, they are not sufficient for representing the complexities of integrative models.

Therefore, the IHM-dictionary extends the definitions in the PDBx/mmCIF dictionary in five significant aspects that address the requirements for archiving integrative models (Vallat et al. 2018).

1. It allows for a flexible model representation with atomic and coarse-grained objects consisting of single and multi-residue spherical beads and three-dimensional Gaussian objects.
2. It supports constitutionally diverse structural assemblies and conformationally diverse ensembles, thereby providing representations for multi-state structural models and models related by time or other order.
3. It captures the spatial restraints derived from different kinds of biophysical techniques, such as CX-MS, SAS methods, EPR spectroscopy, DNA footprinting, mutagenesis, and others. Experimental restraints already captured in the PDBx/mmCIF dictionary and other related extensions are retained and reused where applicable. Several kinds of experimental data provide spatial restraints in the form of distances between atoms or residues (e.g., distances from NMR NOE, FRET, and CX-MS experiments). To address the broad range of experimentally derived distance restraints, the IHM-dictionary includes a general representation of distance restraints between different kinds of features (e.g., atoms, single and multiple residues, contiguous residue ranges) and the corresponding uncertainties associated with these distance measurements. The specifications for different types of spatial restraints are encoded in different data categories within the dictionary. An mmCIF file corresponding to an integrative model derived using restraints from multiple experimental sources will contain several data tables that capture the relevant restraint information. Representation of the spatial restraints in the dictionary enables the visualization of the restraints along with the structural models as well as the validation of integrative models based on the experimental restraints.
4. It provides a generic representation for referencing related data from external resources via stable identifiers, such as accession codes or persistent digital object identifiers (DOIs). This is useful for referencing related data that either lives in an external repository (via stable accession codes) or does not yet have a primary repository (via standard DOIs).
5. It promotes reproducibility by incorporating simplified definitions for the modeling workflow and providing mechanisms to link modeling scripts and software program files.

The IHM-dictionary thus provides a comprehensive set of standardized definitions for representing multi-scale, multi-state, and ordered ensembles of complex macromolecular assemblies. The dictionary has been developed using diverse sets of examples and requirements gathered from the integrative modeling community. Collaborative tools provided by the GitHub platform have been used effectively to gather feedback from the scientific community regarding the definitions in the IHM-dictionary and incorporate their recommendations.

#### Representation of NMR restraints

The contents of the PDBx/mmCIF dictionary (Fitzgerald et al. 2005; Westbrook 2013) grew from a core set of mmCIF definitions describing macromolecular structure and the X-ray diffraction experiment to its current scope through an incremental process of building compatible content extensions (*e.g.*, NMR and 3DEM) in collaboration with community specialists. The development of the IHM-dictionary has followed a similar path by integrating existing definitions in the PDBx/mmCIF dictionary and compatible community extension dictionaries. For example, the IHM-dictionary takes advantage of an existing data dictionary developed to facilitate the programmatic exchange of NMR restraint data, the NEF dictionary (Gutmanas et al. 2015). The IHM-dictionary does not include new definitions for NMR restraints. Rather, definitions from the NEF dictionary are reused to describe NMR restraints used in integrative models.

Creating a consensus representation of NMR restraint data with broad adoption by NMR application developers has proved to be challenging. In part owing to the complexity and diversity of NMR restraint data, neither the NMR-STAR (Markley et al. 2003) representation used by the NMR experimental archive, BioMagResBank (BMRB (Ulrich et al. 2008)), nor the representation adopted by Collaborative Computational Project for NMR (CCPN (Vranken et al. 2005)) gained wide adoption among developers of NMR structure determination and refinement software.

In 2013, a group of NMR experts assembled by the wwPDB, the wwPDB NMR Validation Task Force (VTF), published a set of recommendations for the validation of NMR structure and experimental data archived by the PDB (Montelione et al. 2013). This report included recommendations for restraint-based model-versus-data validation comparing each member of the ensemble of NMR models to the available NMR restraints. Lacking a community consensus representation and format, the wwPDB has historically collected and archived NMR restraint data in native programmatic format. While there have been efforts to retrospectively standardize these native restraint data files using NMR-STAR (Doreleijers et al. 2009), these approaches were not fully automatable and proved difficult to sustain. A Working Group of the wwPDB NMR VTF, including developers of the principal NMR structure determination packages, was subsequently created to revisit the challenges of representing and exchanging NMR restraints and supporting experimental data. In 2015, this Working Group published the first set of recommendations for the NEF dictionary (Gutmanas et al. 2015). In addition to the NMR distance, dihedral, and residual dipolar coupling (RDC) restraint data, the NEF dictionary also includes definitions describing chemical shift and observed spectral peaks. While these data definitions have long been represented in the BMRB NMR-STAR reference dictionary, they are reorganized in the NEF dictionary to simplify their production and exchange by NMR software.

The representation of NMR-specific distance restraints in the NEF dictionary has also informed the development of the representation of generic derived distance restraints for experiment types such CX-MS and FRET in the IHM-dictionary. Work is in progress to build software tools that support the NEF dictionary for the IHM data pipeline.

### The PDB-Dev prototype archiving system

Based on the data standards provided by the IHM-dictionary, we have built a prototype archiving system called PDB-Dev (https://pdb-dev.wwpdb.org) to archive integrative structural models (Burley et al. 2017; Vallat et al. 2018). The integrative structures archived in PDB-Dev conform to the definitions in the IHM-dictionary (Vallat et al. 2018). In order to deposit structures to PDB-Dev, users are required create an account on the PDB-Dev website and upload an mmCIF file that is compliant with the IHM-dictionary. Optionally, supporting files such as images can be included with the deposition. After a structure is deposited, compliance to the IHM-dictionary is checked using software tools built for the PDBx/mmCIF dictionary. If the deposited file is not compliant, communication is initiated with the authors to obtain any missing or incomplete information regarding the deposition. Once a compliant mmCIF file is obtained, the structure is either released immediately or kept on hold until publication. At present, we do not carry out any automated or manual curation of the data or validation of the structural models. The development of a comprehensive deposition, data harvesting, curation and model validation pipeline is the focus of ongoing research.

**Fig. 3:**
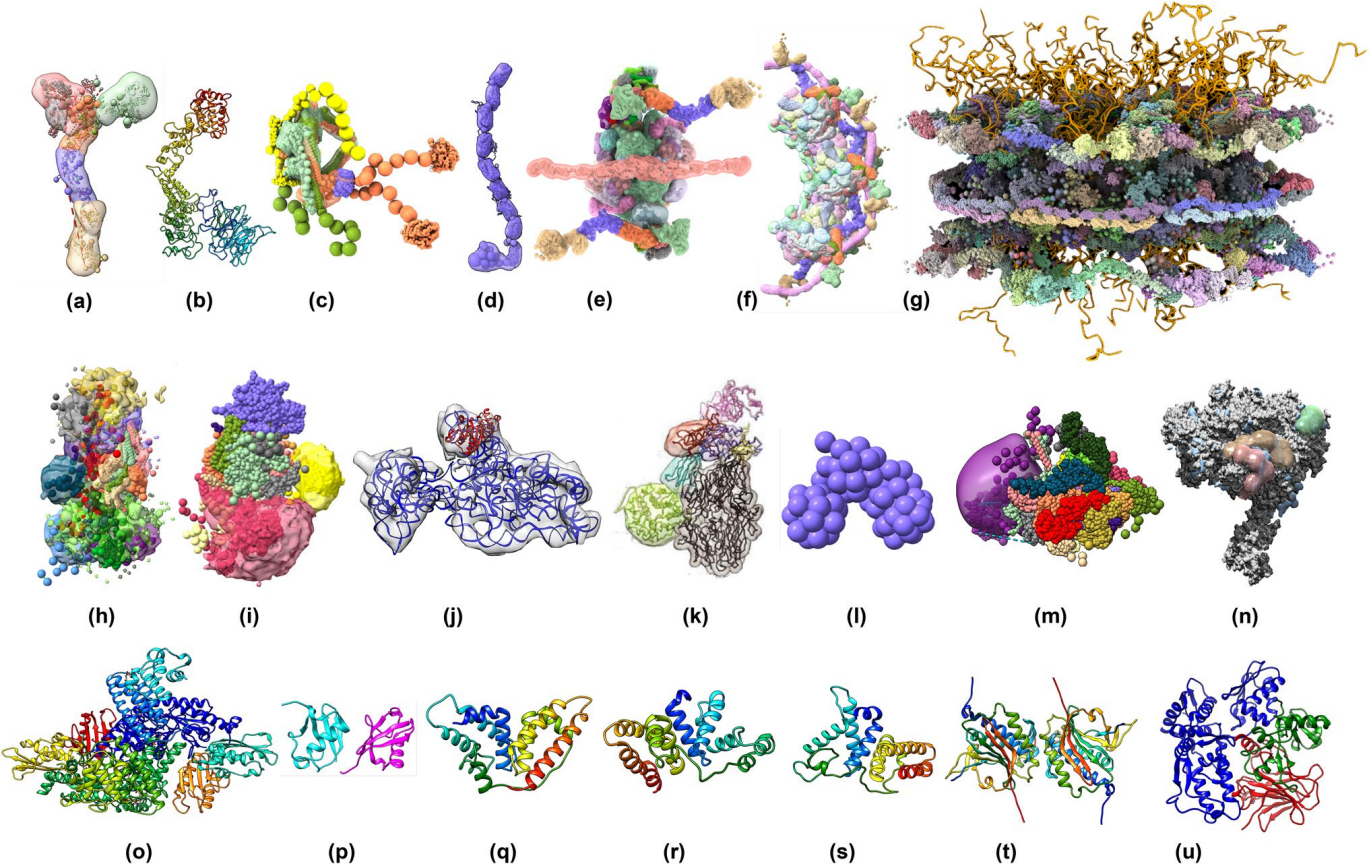
A snapshot of integrative structural models deposited in PDB-Dev. **a** Nup84 sub-complex (PDBDEV_ 00000001 (Shi et al. 2014)), **b** Nup133 sub-complex (PDBDEV_ 00000016 (Kim et al. 2014)), **c** Nup82 sub-complex (PDBDEV_ 00000020 (Fernandez-Martinez et al. 2016)), **d** Pom152 sub-complex (PDBDEV_ 00000017 (Upla et al. 2017)), **e**, **f**, **g** Nuclear pore complex 1-spoke, 3-spokes & 8-spokes (PDBDEV_ 00000010, PDBDEV_ 00000011, PDBDEV_ 00000012 (Kim et al. 2018)), **h** Mediator complex (PDBDEV_ 00000003 (Robinson et al. 2015)), **i** Exosome complex (PDBDEV_ 00000002 (Shi et al. 2015)), **j** 16 s RNA—Methyl transferase A complex (PDBDEV_ 00000014 (van Zundert et al. 2015)), **k** Human complement system C3(H2O) (PDBDEV_ 00000021 (Chen et al. 2016)), **l** Fruit fly chromosome 2L segment (PDBDEV_ 00000008 (Trussart et al. 2015)), **m** Ecm29 protein with 26S proteasome complex (PDBDEV_00000026 (Wang et al. 2017a)), **n** Pol II(G) complex (PDBDEV_00000025 (Jishage et al. 2018)), **o** Mitochondrial cysteine desulfurase complex (PDBDEV_ 00000015 (Cai et al. 2018)), **p** Diubiquitin (PDBDEV_ 00000004 (Liu et al. 2018)), **q**, **r**, **s** Human serum albumin domains A, B & C (PDBDEV_ 00000005, PDBDEV_ 00000006, PDBDEV_ 00000007 (Belsom et al. 2016)), **t** Human Rev7 dimer (PDBDEV_ 00000009 (Rizzo et al. 2018)), **u** E6AP-E6-p53 enzyme–substrate complex (PDBDEV_00000023 (Sailer et al. 2018))

**Fig. 4:**
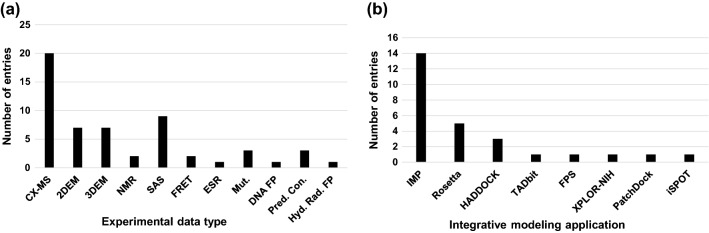
Statistics of current structures in PDB-Dev (including structures released and structures on-hold for publication as of December 6, 2018). **a** Plot of number of entries in PDB-Dev as a function of the type of input experimental restraints. **b** Plot of number of entries in PDB-Dev as a function of the integrative modeling software application used

PDB-Dev currently archives twenty-two integrative structures that have been released along with five additional structures that have been processed and placed on hold for publication. A snapshot of the structures archived in PDB-Dev is shown in Fig. 3. These structures include several macromolecular assemblies, such as the nuclear pore complex (Kim et al. 2018), the mediator complex (Robinson et al. 2015), the exosome complex (Shi et al. 2015), the mitochondrial cysteine desulfurase complex (van Zundert et al. 2015), and others. The integrative structures in PDB-Dev have been obtained by satisfying spatial restraints from different experimental techniques, such as CX-MS, SAS, 2DEM, 3DEM, NMR, EPR, FRET, DNA footprinting, mutagenesis, hydroxyl radical footprinting and predicted contacts from coevolution data (Fig. 4a). Evidently, CX-MS is emerging as a dominant experimental technique to define distance restraints on pairs of cross-linked residues used in integrative modeling, often in combination with 3DEM density maps. Furthermore, the CX-MS field is rapidly evolving to identify novel crosslinking agents and develop better methods for deriving the spatial restraints. Figure 4b shows that the structures archived in PDB-Dev have been modeled using a variety of integrative modeling software tools, including IMP (Russel et al. 2012), Rosetta (Leaver-Fay et al. 2011), Haddock (Dominguez et al. 2003), TADbit (Trussart et al. 2015; Serra et al. 2017), FPS (Kalinin et al. 2012), XPLOR-NIH (Schwieters et al. 2018), PatchDock (Schneidman-Duhovny et al. 2005), and iSPOT (Hsieh et al. 2017). The diversity of software applications that produced the PDB-Dev structures shows that the data standards captured in the IHM-dictionary are generic enough to work with different integrative modeling methods. The model of mitochondrial cysteine desulfurase complex (Fig. 3) built by Haddock (Dominguez et al. 2003) using spatial restraints derived from NMR chemical shift perturbations, SAS, and CX-MS is currently the only example in PDB-Dev that uses NMR data. However, as the integrative modeling methods evolve and the PDB-Dev archive grows, we expect more structures that use restraints derived from NMR experiments to be deposited in PDB-Dev, especially since NMR restraints are inherently amenable to being used in integrative modeling.

The integrative models archived in PDB-Dev can be visualized using the ChimeraX software (Goddard et al. 2018). ChimeraX supports the visualization of multi-scale structural models as well as different types of experimental restraints used in the modeling such as crosslinking distances, 3DEM maps and 2DEM class averages. The images in Fig. 3 have been generated using ChimeraX.

The IHM-dictionary and the PDB-Dev system are under continuous development to address the emerging needs of the integrative modeling community along with a growing range of experimental data types and software applications used to model integrative structures. This effort is carried out in collaboration with the modelers, who provide us with up-to-date examples of integrative models and the associated spatial restraints. We have used these examples as building blocks to develop the IHM-dictionary and the PDB-Dev system. We are also working with the integrative modeling community to build support for the IHM-dictionary within their modeling software, so that these software can easily read and write data files compliant with the IHM-dictionary, thereby streamlining the deposition process of integrative models into PDB-Dev as well as using multiple software programs in one application. The project highlights a concerted community endeavor to create the data standards, develop supporting software tools, and build a prototype system for deposition and archiving integrative structural models.

### Python-ihm library

We have developed the python-ihm software library (https://github.com/ihmwg/python-ihm) to support reading, writing, and managing data files that comply with the IHM-dictionary (Vallat et al. 2018). The python-ihm library implements software support for the IHM-dictionary as a set of Python classes. This implementation allows an integrative model to be represented as a hierarchy of Python objects, and supports reading and writing these hierarchies as IHM-dictionary-compliant mmCIF data files, as well as binary representations such as BinaryCIF (Sehnal 2016). It is available under a permissive open source license, and is designed to be used either standalone or as part of an integrative modeling package. By providing a software implementation of the dictionary, developers of integrative modeling software are relieved of the burden of developing their own support for IHM-dictionary; this service should lower the barrier to entry to PDB-Dev (Burley et al. 2017; Vallat et al. 2018). For example, both IMP (Russel et al. 2012) and Haddock (Dominguez et al. 2003) already use python-ihm to output their models in a format compliant with the IHM-dictionary for deposition in PDB-Dev. Furthermore, the ChimeraX visualization software (Goddard et al. 2018) uses the python-ihm library to support visualization of integrative models archived in PDB-Dev.

### Challenges and future perspectives

In the last 4 years, there has been substantial progress in creating the framework for archiving integrative structure models. The creation of an extensible dictionary has made this archival possible as has the development of the PDB-Dev test platform that allows for prototyping an archiving system. There are considerable challenges ahead. The first is the creation of standards for all the experimental methods that contribute restraints to the modeling. Achieving this goal will require that each experimental community reach consensus on their own standards. The second is to find a mechanism to exchange these data among all the relevant communities and with the PDB archive. The last and most difficult challenge is to come up with methods to validate each model so that it will be possible for users of these models to understand their limits. Meeting these challenges will require further scientific research, technology development and implementation, and most of all a spirit of collaboration and cooperation among the very heterogeneous communities.
